# Influence of Thyroid Peroxidase Antibodies Serum Levels in Graves' Disease: A Retrospective Cohort Study

**DOI:** 10.7759/cureus.40140

**Published:** 2023-06-08

**Authors:** Maria L Guia Lopes, Carlos Tavares Bello, José P Cidade, Clotilde Limbert, Joao Sequeira Duarte

**Affiliations:** 1 Department of Endocrinology, Hospital Egas Moniz, Centro Hospitalar Lisboa Ocidental, Lisbon, PRT; 2 Department of Endocrinology, Hospital da Luz Lisboa, Lisbon, PRT; 3 Department of Internal Medicine, Hospital Egas Moniz, Centro Hospitalar Lisboa Ocidental, Lisbon, PRT; 4 Department of Intensive Care, Hospital de São Francisco Xavier, Centro Hospitalar Lisboa Ocidental, Lisbon, PRT; 5 Department of Physiology, Nova Medical School, New University of Lisbon, Lisbon, PRT

**Keywords:** graves' disease, thyroid peroxidase antibodies, relapse, remission, antithyroid drugs

## Abstract

Purpose

Graves' disease (GD) is an autoimmune disorder caused by the presence of antibodies to the thyroid stimulating hormone (TSH) receptor (TRAbs), usually presenting with clinical signs of hyperthyroidism. Previous evidence suggests that higher serum levels of thyroid peroxidase antibodies (TPOAbs) may lead to more sustained remission of hyperthyroidism after treatment with antithyroid drugs (AT). However, doubts about the influence of TPOAbs in Graves' disease outcomes still remain.

Methods

A retrospective, unicenter cohort study was performed. All patients with GD (TRAbs > 1.58U/L), biochemical primary hyperthyroidism (TSH < 0.4 µUI/mL), and TPOAbs measurement at diagnosis, treated with AT between January 2008 and January 2021, were included for analysis.

Results

One hundred and forty-two patients (113 women) with a mean age of 52 ± 15 years old were included. They were followed up for 65.4 ± 43.8 months. TPOAbs positivity was present in 71.10% (n=101) of those patients. Patients were treated with AT for a median of 18 (IQR (12; 24)) months.

Remission occurred in 47.2% of patients. Patients with remission presented with lower TRAbs and free thyroxine (FT4) levels at the diagnosis. (p-value <0.001, p-value 0.003, respectively). No association was found in the median TPOAbs serum levels of patients who remitted and those who maintained biochemical hyperthyroidism after the first course of AT.

Relapse of hyperthyroidism occurred in 54 patients (57.4%). No difference was found in TPOAbs serum levels regarding the patient's relapse. Moreover, a time-based analysis revealed no differences in the relapse rate after 18 months of AT therapy between patients with and without TPOAbs positivity at the diagnosis (p-value 0.176).

It was found a weak positive correlation (r=0.295; p-value <0.05) between TRAbs and TPOAbs titters at the moment of Graves' diagnosis.

Conclusion

In this study, a correlation between TRAbs measurements and TPOAbs titter was described, although no significant association was found between the presence of TPOAbs and the outcomes of patients with GD treated with AT. These results do not support the use of TPOAbs as a useful biomarker to predict remission or relapse of hyperthyroidism in GD patients.

## Introduction

Graves' disease (GD) is an immune-mediated disorder characterized by primary hyperthyroidism, ophthalmopathy (Graves' ophthalmopathy), and/or pretibial myxedema. It represents the most common etiology of primary hyperthyroidism (80% of cases), and it is more common in the fifth and sixth decades with a male-to-female ratio of 1:10 [1-3].

The thyroid stimulating hormone (TSH)-receptor antibodies (TRAbs) are the main biochemical hallmark of GD. These antibodies lead to overstimulation of the TSH receptor with a subsequent increase in thyroid hormone production. Moreover, elevated titers of TRAbs underlie the majority of the clinical changes observed in these patients, and their detection has high clinical value due to high diagnostic sensitivity and specificity [4].

In the majority of cases, Graves' hyperthyroidism is treated by decreasing thyroid hormone synthesis using antithyroid drugs (AT). Thionamides inhibit the coupling of iodothyronines, as well as the function of thyroperoxidase, reducing oxidation and the organification of iodide into thyroid hormones [2,5]. Patients with GD are usually treated with AT for a period of 12-18 months, and remission rates can be as high as 50% after the first course of AT [6]. The TRAbs serum levels are an important clinical determinant before the decision of these treatments' suspension [2,7].

Thyroid peroxidase antibodies (TPOAbs) clinical relevance is well established as a diagnostic biomarker of Hashimoto's thyroiditis. In those cases, they seem to represent a secondary response to thyroid injury and are not thought to cause disease themselves [8,9]. Nevertheless, these antibodies are also frequently found in patients with GD (50-90%) and less often in healthy individuals, creating a discrepancy between different experts' opinions and raising doubts about their pathological role in these diseases [9-12]. 

The main aim of this study is to evaluate the potential role of TPOAbs in predicting GD's remission and its correlation with clinical hyperthyroidism relapse. The authors hypothesize that there may be a positive association between the presence of TPOAbs positivity and the outcomes of patients with GD treated with AT.

## Materials and methods

Study cohort and sampling

A retrospective cohort study was conducted at the department of endocrinology of a tertiary referral endocrinological center (Hospital de Egas Moniz, Centro Hospitalar de Lisboa Ocidental, Lisboa). Data were collected from patients enrolled between January 2008 and January 2021 using the department's electronic database. The patient's anonymity was maintained during the length of the study.

All patients eligible met the following inclusion criteria: age above 18 years, GD's diagnosis, and absence of missing data on TPOAbs measurement at the moment of diagnosis. A follow-up period of at least 12 months after ATD treatment discontinuation was also considered necessary. Pregnant women were not included.

The diagnosis of Graves' disease was assumed in the presence of the following criteria: clinical signs of hyperthyroidism (namely tachycardia, anxiety, weight loss, and diarrhea), TRAbs >1.58 U/L, and biochemical hyperthyroidism (TSH <0.4 µUI/mL) following the criteria defined by the European Thyroid Association [2]. TPOAbs positivity was assumed if the patient's serum values were above 35 IU/mL.

In line with American Thyroid Association recommendations, remission was considered if there was a proven absence of clinical and biochemical signs of hyperthyroidism (including serum normal levels of TRAbs) until 12 months after AT withdrawal [6]. Patients with hyperthyroidism relapse before 12 months or with continuous hyperthyroidism despite AT were considered the "non-remission" group.

Relapse was established with the recurrence of clinical and biochemical signs of hyperthyroidism after at least one month of AT suspension. When multiple relapses occurred within a patient, only the first course of AT was considered for the analysis.

Patients were treated with antithyroid drugs (propylthiouracil or methimazole). The drug of choice and its posology were based on the assistant physician's decision while taking into account the patient's preferences. The moment of AT suspension was determined by the patient's assistant physician.

The following patient variables were collected: age, gender, the presence of Graves' ophthalmopathy, date of remission, date of recurrence, definitive treatment modality (when considered), and the following serum biomarkers' levels at admission: TSH, free thyroxine (FT4), free triiodothyronine (FT3), TPOAb and TRAbs; and after the end of the first course with AT: TSH, free thyroxine (FT4), free triiodothyronine (FT3) and TRAbs.

The Ethics Committee of the Hospital Egas Moniz, Centro Hospitalar Lisboa Ocidental, approved the study. Considering the observational nature of study and the anonymity of the data collected, the ethic committee dismissed the need of written informed consent. All details and data collected that might disclose the subjects under the study were omitted or anonymized.

Thyroid function assessments

Thyroid function tests were measured with an automated direct chemiluminescent method (normal range: TSH 0.27 to 4.2 μIU/mL, free T4 12.0 to 22.0 pmol/L, and free triiodothyronine (T3) 3.10 to 6.80 pmol/L). Thyroid autoantibody assays were also performed by an automated chemiluminescent method; normal range: TRAb 0 to 1.58 U/L and TPOAb <35 UI/mL).

Statistical analyses

All Gaussian distributed variables were expressed as mean (SD), and nonnormally distributed variables as median (interquartile range (IQR)). Continuous variables were expressed as numbers and percentages. Missing values were handled by patients' exclusion in order to eliminate any associated missing at random bias.

The Chi-squared test was used for categorical variables, and the t-test and Kruskal-Wallis were used on continuous variables for statistical assessment of outcomes between groups. Kaplan-Meier survival curve and log-rank test were also obtained to ascertain and compare survival between groups.

In all the hypothesis tests, a p-value of less than 0.05 was considered for statistical significance and usual confidence intervals of 95% were chosen. All statistical analyses were performed using SPSS version 21 (IBM Inc., Armonk, New York).

## Results

One hundred and forty-two patients were eligible for statistical analysis. The main demographic characteristics depicted a mean age of 52 ±15 years and a female predominance (79.6% of patients). Patients were followed up for a mean of 65.4 ± 43.8 months. Patients' biochemical baseline characteristics at the diagnosis and after the first course of AT treatment are summarized in Table 1.

**Table 1:** Patients' biochemical baseline biomarkers at the diagnosis and after the first course of AT treatment TSH - thyroid stimulating hormone, TRAbs - thyroid stimulating hormone receptor antibodies, TPOAbs - thyroid peroxidase antibodies, AT - antithyroid drug

Biochemical biomarkers	At diagnosis	After treatment
TSH (µUI/mL, median, IQR)	0.01 (0.01; 0.02)	2.04 (0.81; 3.80)
Free T4 (pmol/L, mean, SD; median, IQR)	30.00 (18.87; 48.57)	13.18 (11.15; 16.48)
Free T3 (pmol/L, median, IQR)	8.94 (5.79;18.35)	4.98 (4.34; 5.48)
TRAbs (U/L, median, IQR)	7.12 (3.50; 20.52)	1.10 (0.80; 2.62)
TPOAbs (IU/mL, median, IQR)	180.50 (22.85; 600.00)	-
TPOAbs positivity, n (%)	101 (71.1%)	-

At diagnosis, TPOAbs positivity was verified in the majority of patients (71.1%), and its serum levels were positively correlated with TRAbs serum levels (Spearman correlation coefficient, r=0.288, p<0.001). Patients were treated with AT (thiamazole and propylthiouracil) for a median of 18 months (IQR 12; 24). After the first course of AT, remission occurred in 47.2% of patients. Higher TRAbs serum levels at the diagnosis were weakly correlated with a longer hyperthyroidism state during AT therapy (Spearman's correlation coefficient, r=0.25; p=0.015).

Patients with remission presented with lower TRAbs and FT4 levels at the diagnosis. In what concerns patients' remission, no difference was found in the median TPOAbs levels between those who remitted and those who did not (183 (815; 773) IU/mL versus 180 (26; 542) IU/mL, respectively, p=0.825). Patients' demographic characteristics and analytical biomarkers at the diagnosis, regarding GD remission after AT treatment, are resumed in Table 2.

**Table 2:** Patients' demographic characteristics and analytical biomarkers at the diagnosis, regarding GD remission after AT treatment ¥ p-values were determined using two-tailed unpaired Student t-test and Kruskal-Wallis H test TRAbs - thyroid stimulating hormone receptor antibodies, TPOAbs - thyroid peroxidase antibodies, AT - antithyroid drug, TSH - thyroid stimulating hormone

Demographic and analytical variables	Remission (n=67)	No remission (n= 75)	p-value^¥^
Age (years, mean, SD)	50.3 (±14.0)	53.1 (±15.4)	0.338
TRAbs (U/L, median, IQR)	4.6 (2.8;14.2)	9.4 (4.6;25.4)	<0.001
TPOAbs (IU/mL, median, IQR)	183 (815;773)	180 (26;542)	0.825
TPOAbs positivity (yes, n)	46	55	0.539
TSH (µUI/mL, median, IQR)	0.010 (0.008;0.02)	0.010 (0.001;0.02)	0.250
T4L (pmol/L, median, IQR)	27.5 (17.4;43.4)	38.5 (21.0;56.1)	0.028
T3L (pmol/L, median, IQR)	8.0 (5.8;14.2)	11.6 (5.7;20.0)	0.380

**Figure 1:**
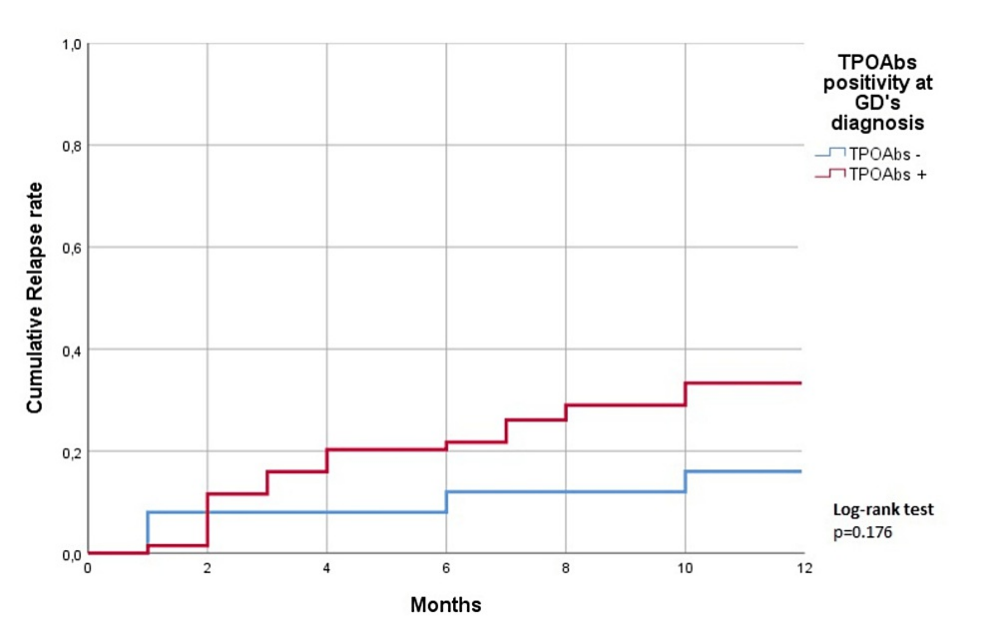
Relapse rate after 12 months of AT withdrawal, between patients with TPOAbs positivity (TPOAbs +) and without TPOAbs positivity (TPOAbs -) at diagnosis TPOAbs - thyroid peroxidase antibodies, AT - antithyroid drug

Additionally, using a time-based event analysis, no difference was found in the relapse after 12 months of AT withdrawal, between patients with TPOAbs positivity (TPOAbs +) and without TPOAbs positivity (TPOAbs -) at the diagnosis (p=0.176) (Figure 1).

Relapse of GD hyperthyroidism occurred in the majority of the patients (n=54; 57.4%;) after a median follow-up of 11 (4; 26) months after AT withdrawal. No difference was found in TRAbs serum levels between relapsing and non-relapsing patients (p=0.180). 

In the specific subset of patients with hyperthyroidism relapse before 12 months of AT withdrawal, higher serum levels of TRAbs were found at the diagnosis (14.0 (4.5; 39.3) vs. 3.5 (1.8; 10.3), p=0.001). Higher TRAbs titters at the diagnosis were correlated with an earlier relapse after treatment withdrawal (Spearman's correlation coefficient, r: 0.512; p<0.001).

Patients' demographic characteristics and analytical biomarkers at the diagnosis, regarding GD relapse after AT treatment, are depicted in Table 3. No difference was found in TPOAbs serum concentration, determined at the diagnosis, between relapsing and non-relapsing patients (247.00 (82.33; 610.00) IU/mL vs. 195.50 (15.00; 933.00), respectively, p=0.353). TPOAbs positivity was also not different between those groups (Table 3).

**Table 3:** Patients' demographic characteristics and analytical biomarkers at the diagnosis, regarding GD relapse after AT treatment ¥ p-values were determined using two-tailed unpaired Student t-test and Kruskal-Wallis H test TRAbs - thyroid stimulating hormone receptor antibodies, TPOAbs - thyroid peroxidase antibodies, AT - antithyroid drug, TSH - thyroid stimulating hormone

Demographic and analytical variables	Relapse (n=54)	No relapse (n=40)	p-value^¥^
Age (years, mean, SD)	52.20 (± 13.34)	49.62 (± 13.53)	0.551
TRAbs (U/L, median, IQR)	6.00 (2.98; 21.93)	6.35 (3.13; 16.81)	0.180
TPOAbs (IU/mL, median, IQR)	247.00 (82.33; 610.00)	195.50 (15.00; 933.00)	0.353
TSH (µUI/mL, median, IQR)	0.01 (0.01; 0.02)	0.01 (0.01; 0.02)	0.178
T4L (pmol/L, median, IQR)	30.00 (18.85; 48.15)	27.75 (17.75; 46.03)	0.634
T3L (pmol/L, median, IQR)	8.97 (5.77; 17.98)	8.65 (6.02; 16.60)	0.998

## Discussion

GD hyperthyroidism usually resolves within the first 12-18 months of therapy with AT [2,6]. Hyperthyroidism relapse is more common within the first year after stopping treatment (almost 55% of patients) [13,14]. Therefore, high-level evidence is crucial to define potential predictive GD biochemical markers in order to promptly identify patients with a higher probability of GD relapse. This could facilitate the promotion of individually-tailored treatment approaches and, consequentially, the improvement in patients' long-term outcomes.

In that regard, it is known that higher TRAbs levels at diagnosis correlate with worse clinical outcomes after treatment [15,16]. However, the role of other thyroid antibodies in GD outcomes, specifically TPOAbs, has not been established.

In a previous study, Schott et al. [17] aimed to ascertain the prognostic value of different biomarkers in GD and did not find any role of TPOAb levels at diagnosis in the remission rate of these patients. However, at the time, the authors proposed a predictive remission model using TRAbs and TPOAbs serum levels that performed reasonably in the prediction of poor disease outcomes [17]. In our study, data supported a clear correlation between TRAbs and TPOAbs titers at the diagnosis. Nonetheless, similarly to that study, no association between TPOAbs serum titters or TPOAbs positivity and remission rates was described.

Moreover, in what concerns TPOAbs serum levels and relapse in GD patients, the evidence collected in our study was different from that already available in previous studies. Muir et al. [18] described that the presence of lower TPOAbs levels at GD diagnosis was more frequent in patients who relapsed after a course of AT. They proposed that TPOAbs positivity was negatively correlated with relapsing rates and could reflect some grade of lymphocytic infiltration and subsequent loss of thyroid function [18-20]. On the other hand, Hamada et al. [19] showed an association between TPOAbs positivity and higher relapse rates in GD patients [19,21]. This was supported by another Korean study [22], which concluded that patients with a persistent increase in TPOAb titers during treatment had shorter relapse-free time, independently of thyroid function or TRAbs titers. In our study, there was no evidence of an association between TPOAbs serum concentration and GD relapse rate. Moreover, TPOAbs positivity was not different between relapsing and non-relapsing patients.

Our results seem to sustain that TPOAbs levels might be an indicator of GD autoimmune activity and a good surrogate of TRAbs serum levels at the diagnosis. However, it does not seem to retain a good predictive value of remission or relapse rates in these patients. Therefore, our data do not support the widespread use of this biomarker as a prognostic indicator or reliable criteria to guide AT treatment and its suspension.

Our study convenes several strengths. It is composed of a large cohort of GD patients with a consistent clinical follow-up without significant missing data or study drop-outs. However, it is not without limitations. It is a retrospective study, and data concerning TPOAbs determination during treatment or at the GD relapse, were not consistently collected. Furthermore, it encompasses different AT posology and protocols, considering that those variables were determined by the patients' assistant physicians. Finally, data regarding thyroid volume (due to the absence of full sonographic data) was also not registered.

## Conclusions

In this study, there was no evidence of a relationship between TPOAbs levels at the diagnosis and GD remission and relapse rates. Given the high prevalence of TPOAbs positivity and its documented low specificity, our study does not support the use of TPOAbs levels to predict GD outcomes. However, because TPOAbs titers correlate with TRAbs levels, they may be associated with the disease's immune burden, and more studies are needed to conclusively define its role in GD.
